# Climate-responsive DNA methylation is involved in the biosynthesis of lignin in birch

**DOI:** 10.3389/fpls.2022.1090967

**Published:** 2022-12-02

**Authors:** Bowei Chen, Yile Guo, Xu Zhang, Lishan Wang, Lesheng Cao, Tianxu Zhang, Zihui Zhang, Wei Zhou, Linan Xie, Jiang Wang, Shanwen Sun, Chuanping Yang, Qingzhu Zhang

**Affiliations:** ^1^ State Key Laboratory of Tree Genetics and Breeding, Northeast Forestry University, Harbin, China; ^2^ College of Life Science, Northeast Forestry University, Harbin, China

**Keywords:** lignin, climate change, DNA methylation, *BpNST1/2* and *BpSND1*, secondary metabolite

## Abstract

Lignin is one of the most important secondary metabolites and essential to the formation of cell walls. Changes in lignin biosynthesis have been reported to be associated with environmental variations and can influence plant fitness and their adaptation to abiotic stresses. However, the molecular mechanisms underlying this association remain unclear. In this study, we evaluated the relations between the lignin biosynthesis and environmental factors and explored the role of epigenetic modification (DNA methylation) in contributing to these relations if any in natural birch. Significantly negative correlations were observed between the lignin content and temperature ranges. Analyzing the transcriptomes of birches in two habitats with different temperature ranges showed that the expressions of genes and transcription factors (TFs) involving lignin biosynthesis were significantly reduced at higher temperature ranges. Whole-genome bisulfite sequencing revealed that promoter DNA methylation of two NAC-domain TFs, *BpNST1/2* and *BpSND1*, may be involved in the inhibition of these gene expressions, and thereby reduced the content of lignin. Based on these results we proposed a DNA methylation-mediated lignin biosynthesis model which responds to environmental factors. Overall, this study suggests the possibility of environmental signals to induce epigenetic variations that result in changes in lignin content, which can aid to develop resilient plants to combat ongoing climate changes or to manipulate secondary metabolite biosynthesis for agricultural, medicinal, or industrial values.

## Introduction

Climate change includes the instantaneous and rapid alterations of numerous important environmental parameters that regulate the dynamics of the ecosystem, such as temperature and precipitation. These rapid changes may have direct and indirect effects on plants *via* alterations in plant secondary metabolism (Christensen and Christensen, 2007; Bita and Gerats, 2013; Rienth et al., 2021). Lignin is one of the most important secondary metabolites and is essential to the formation of cell walls. Changes in lignin biosynthesis can influence plant fitness and their adaptation to environment (Liu et al., 2018). For example, a lignin-deficient mutant of *Arabidopsis* was found to have a significantly lower seed germination rate compared to the wild type, hypothetically due to an increase of sensitivity to unfavorable environments (Liang et al., 2006). Moreover, previous studies have shown that the lignin biosynthesis is highly responsive to environmental changes (Moura et al., 2010; Cesarino, 2019; Gu et al., 2020). Under drought and salt stresses, increased lignin can reduce plant cell wall water penetration and transpiration, which helps to maintain cell osmotic balance and protect membrane integrity (Monties and Fukushima, 2001; Chun et al., 2019). When plants are exposed to an excess of light, an increased level of lignin content is observed, which may prevent the accumulation of hydrogen peroxide and thus protect the cellular damage from the formation of reactive oxygen species (ROS) (Chen et al., 2002). Similarly, increased lignin content can reduce damage from ultraviolet (UV)-B radiation in plants (Rozema et al., 1997). In addition, changes in lignin content are also found to be related to cold acclimation (Wei et al., 2006; Zeng et al., 2016). One possible reason is that the altered ratio of syringyl (S) to guaiacyl (G) subunits affects cell wall rigidity and water permeability, thus increasing plant resistance to cold temperatures (Zhao et al., 2013). Overall, these previous findings indicate that lignin biosynthesis is tightly associated with environmental conditions. Deciphering the molecular basis underlying this association is thus crucial to understanding plants adaptation and evolution and to promoting plants conservation and breeding.

DNA methylation (5-methylcytosine) is an important regulatory mechanism by which plants respond to environmental fluctuations (McCaw et al., 2020; Sun et al., 2022). In plants, DNA methylation occurs in three sequence contexts: CG, CHG, and CHH, where H is any nucleotide except G. Maintenance DNA methylation in symmetrical CG and CHG contexts is executed by DNA METHYLTRANSFERASE 1 (MET1) and CHROMOMETHYLASE 3 (CMT3), respectively. Asymmetric CHH methylation is maintained by CHROMOMETHYLASE 2 (CMT2) and the 24nt-siRNA involved RNA-directed DNA methylation (RdDM) pathway (Law and Jacobsen, 2010; Zemach et al., 2013; Stroud et al., 2014). Changes in DNA methylation in response to altered environmental conditions may facilitate plant adaptation *via* their effects on gene expressions. For example, in *B. gymnorhiza*, genome-wide CHH hypermethylation induced by increased environmental salinity can enhance several salt-responsive genes expression, such as *Bg70* and BURP Domain Containing protein 3 (*BgBDC3*) (Miryeganeh et al., 2022). In maize, genome-wide demethylation enables the activation of stress-responsive genes under cold stresses (Steward et al., 2002). In natural populations, genome-wide CHH methylation variations are associated with the changes of temperature seasonality in *Arabidopsis* accessions (Shen et al., 2014). CG methylation variations, which affects dehydration-responsive genes in oak, are found to be significantly associated with the mean maximum temperature of the warmest month (Gugger et al., 2016). Together, these results provide evidence that epigenetic variation can be an important molecular mechanism that influences plant adaptations to the changing climates.

Since DNA methylation and lignin production can both be adaptive responses to external environments, we hypothesized that DNA methylation is likely to respond to environmental fluctuations by participating in lignin regulation. This was based on two lines of evidence. First, previous studies have shown that the variations of DNA methylation are, at the same time, associated with the biosynthesis of secondary metabolism and with the environmental gradients. For example, in birch, variations in methylation of the *bHLH9* promoter are found to affect the biosynthesis of betulin and be significantly associated with local temperatures and precipitations (Wang et al., 2021). In wheat, DNA methylation of genes involved in carbon metabolism or fatty acid metabolism contributes to male spikelet sterility in a temperature-sensitive genic male sterile (TGMS) line, BS366, in response to cold (Liu et al., 2021). Further, DNA methylation levels involved in metabolic processes are significantly associated with high temperatures in rice (Li et al., 2022). These results provide the evidence that the regulatory role of DNA methylation in plant adaptation can be mediated by its effects on secondary metabolisms.

Second, lignin biosynthesis is a very complex network, involving many enzymes and transcription factors (TFs). Lignin monomer synthesis is mainly composed of phenylpropane metabolic pathways and downstream synthases (Xie et al., 2018). The classic phenylpropane metabolic pathways are primarily composed of phenylalanine ammonia-lyase (PAL), cinnamate-4-hydroxylase (C4H), and 4-coumarate: CoA ligase (4CL), contributing to the conversion of phenylalanine into *p*-coumaroyl-CoA (Fraser and Chapple, 2011; Wang et al., 2018). In addition to these three enzymes, some other phenylpropanoid biosynthetic enzymes are responsible for the sequential conversion of intermediate/downstream products of the phenylpropane metabolic pathway into lignin monomers (Raes et al., 2003; Yoon et al., 2015). The polymerization of lignin monomers is then carried out by peroxidase (POD) and laccase (LAC) (Liu et al., 2011; Alejandro et al., 2012). Moreover, the transcriptional regulation of lignin biosynthesis genes is mainly well-known for the first-layer NAC-domain TFs (Kubo et al., 2005; Zhong et al., 2007; Fang et al., 2020), and the second-layer MYB-domain TFs (Ko et al., 2014; Nakano et al., 2015; Xiao et al., 2021). This complexity can offer more chances in which DNA methylation could be involved. As an example, we recently found that a transcription factor, *CmMYB6*, that regulates another downstream branch of phenylpropane metabolic pathways, can have methylation variations in the promoter which regulates anthocyanins biosynthesis (Tang et al., 2022). However, whether DNA methylation is involved in lignin biosynthesis and this relation, if exist, would contribute to environmental adaptation in natural conditions are still unclear.

In this study, to investigate the relation of DNA methylation with lignin and its role in plant adaptation, Chinese birch (*Betula platyphylla Suk.*), which is widely distributed in the northeast China, was selected as the target species (Geng et al., 2022). Lignin content, methylome and transcriptome profiles of genes involved in the lignin biosynthesis pathway were assessed to uncover a possible molecular regulatory mechanism and to test their relations with climatic factors. We found that the lignin content of natural birch was significantly negatively correlated with local temperature ranges. Nearly all critical lignin biosynthesis genes were expressed at significantly higher levels in birch trees grown at low temperature ranges than at high temperature ranges. This differential expression was likely regulated by two master switches of lignin activators, *BpNST1/2* and *BpSND1*, whose expressions were likely regulated by differential methylation of the promoters between birches grown in different temperature ranges. In summary, our results support the hypothesis that DNA methylation can be involved in responses to climate changes *via* its regulation on plant secondary metabolites and can further provide useful information about how to manage the white birch to efficiently utilize lignin from natural birch sources.

## Materials and methods

### Study area and sampling

To ensure that the sampling covered the entire distribution area of birch (*Betula platyphylla*) in the northeast China (119.91°-133.75°E, 41.32°-53.15°N), four representative regions with distinct climates were selected: Changbai Mountain, Xiaoxing Anling, Daxing Anling and Sanjiang Plain. The landscape was characterized by plains and mountains with elevations ranging from 55.2 to 995.0 m. The Annual Mean Temperature (*bio1*) varied between -4.71°C and 5.15°C, and the Temperature Annual Range (*bio7*) differed from 43.34°C to 61.56°C. Precipitation primarily occurred during the crop-growing season (from April to August), and the Annual Precipitation (*bio12*) ranges from 420 to 935 mm.

In the summer of 2019, cores were collected from 60 sites with naturally growing 20-30 years old birch trees (**Supplementary Figure S1** and **Supplementary Table S1**). Among them, 17 sampling sites were distributed in Changbai Mountain, 12 were distributed in Xiaoxing Anling, 27 were distributed in Daxing Anling, and 4 were distributed in Sanjiang Plain. We sampled one individual from each sampling site and used the abbreviated name of sampling sites to indicate each sample. For example, “Sl”, “Fhs”, “Ame” and “Alh” were used to refer to the sample from “Shulan”, “Fenghuangshan”, “Amuer”, and “Alihe” sampling sites, respectively. For each individual, cores were collected at 15-20 cm in diameter at breast height (DBH, 1.3 m above the ground) (Guo et al., 2017). Collected fresh leaves and cores were immediately stored in liquid nitrogen and dry sealed tubes, respectively, for subsequent experiments.

### Determination of the tree age

Collected cores were first fixed in wooden troughs and left to dry completely at room temperature (20°C) for at least 7 days. Subsequently, the 400-grit and 600-grit sandpaper were used to perform rough grinding and fine grinding in turn until the surface of the cores was smooth and the boundaries of tree ring were exposed. Finally, the treated cores were calibrated under a microscope to determine the age of each birch.

### Determination of the lignin content

To determine the lignin content in each birch individual, the Soxhlet extraction method was adopted in this study (Arni, 2018; Saha et al., 2019). The first step was to extract the wood powder. Dried cores were ground with a 40-grit sandpaper, and the resulting wood powder was stored in a Soxhlet extractor. The Soxhlet extractor was then connected to an extraction device containing 300 ml of extraction solution (ethanol: benzene = 1:2) for 6 hours and transferred to a fume hood to air-dry for at least 48 hours.

The second step was to measure the weight of dry wood powder. Extracted wood powder (0.0500~0.0505 g, recording the weight as *W1* (g)) was placed in an oven at 105°C for at least 8 hours. After that, the dried wood powder was transferred to a vacuum dryer and vacuumed for about 10 min, then transferred to dry conditions and naturally cooled for 1 hour. The final weight was recorded as *W2* (g). Moisture content and dry wood powder were calculated as follows:


$$\text{moisture content }\left(\text{MC}\right)=\frac{\left(W2-W1\right)}{W2}\;\times \;100\%$$

dry wood powder (DWP)=(1−MC)× W2

The third step was to measure the content of lignin. Extracted wood powder (0.0500~0.0505 g) was first acid-hydrolyzed by mixing with 1.5 ml of 72% H_2_SO_4_ solution in a 10-ml glass tube for 90 min. Each sample was transferred into a 100-ml glass bottle, supplemented with ultrapure water to 36 ml, then sealed and autoclaved at 121°C for 90 min. After cooled to room temperature and suction filtered, 10 ml of filtrate was measured at 205 nm using a UV spectrophotometer (*W3*). The remaining solid particles in the bottles were washed with 200 ml of ultrapure water and stored in a crucible. After drying in an oven at 105°C for 8 h, the weight was recorded as *W4* (g). Three independent replicates were performed for each sample. Lignin content was calculated as follows:


$$\text{lignin content}=\left(\frac{W3\;\times \;36}{\text{DWP }\times \;1.1}\;+\;\frac{W4}{\text{DWP}}\right)\times \;100\%$$

### Collection of climate data

The 19 climatic variables (*bio1* ~ *bio19*) used in this study were extracted from the WorldClim 2.0 database using the R package “Raster” (Fick and Hijmans, 2017) based on the recorded longitude and latitude information of the sampling sites (**Supplementary Tables S1**, **S2**). Specifically, *bio5*, *bio8* and *bio10* are climatic variables related to summer temperatures, *bio6*, *bio9* and *bio11* are related to winter temperatures, *bio2*, *bio3*, *bio4* and *bio7* are related to temperature ranges, *bio13*, *bio16* and *bio18* are related to summer precipitations, and *bio14*, *bio17* and *bio19* are related to winter precipitations.

### Identification of synthases genes and TFs involved in lignin biosynthesis

15 lignin biosynthesis synthases in birch, namely *BpPAL1*, *BpPAL3*, *BpC4H2*, *BpC4H3*, *Bp4CL2*, *BpHCT1*, *BpC3H1*, *BpCCoAOMT2*, *BpCCR1*, *BpCCR4*, *BpF5H2*, *BpCOMT9*, *BpCOMT17*, *BpCAD9* and *BpCAD12*, were acquired from Chen et al. (2020). 18 TFs involved in regulation of lignin biosynthesis in birch, namely *BpNST1*/*2*, *BpSND1*, *BpVND6*/*7*, *BpMYB46*/*BpMYB83*, *BpMYB61*, *BpMYB75*, *BpMYB103*, *BpMYB3*/*4*/*7*/*32*, *BpMYB20*/*43*/*85* and *BpMYB15*/*58*/*63*, were identified by BLAST alignment and a phylogenetic tree with known lignin-regulated TFs in *Arabidopsis* (Xiao et al., 2021).

### Next-generation sequencing and data processing

We collected the fresh mature leaves of Sl (127.12˚E, 44.20˚N, 18.5˚C), Fhs (127.98˚E, 44.22˚N, 19.0˚C), Ame (123.34˚E, 52.86˚N, 15.5˚C), and Alh (123.69˚E, 50.63˚N, 17.0˚C) in June of 2019. The collected leaves were immediately stored in liquid nitrogen in sampling sites, and used to assess the methylome and transcriptome profiles of genes involved in the lignin biosynthesis pathway. Previous studies have shown that lignin content and the methylation as well as expression levels of related biosynthetic and regulatory genes in different tissues may be the same trend (Barber and Mitchell, 1997; Davey et al., 2004; Akgul et al., 2007; Zagoskina et al., 2007; Wang et al., 2016; Wang et al., 2021). We, therefore, used leaves for both methylome and transcriptome experiments instead of stems due to technical obstacles.

Genomic DNA was extracted from fresh leaves using the CTAB method (Pan et al., 2006). DNA was recovered from a sizing gel to select for fragment size, and PCR amplification was performed to construct the MethylC-seq library, and finally, an Illumina Hiseq 2000 sequencer was used for PE150 sequencing. Raw HiSeq data was filtered using Fastp, and the clean reads were mapped to the reference genome (*Betula pendula*) using BSMAP software with default parameters (Li and Li, 2009; Salojarvi et al., 2017; Chen et al., 2018). Methylated cytosines were called using a Python script in BSMAP. A WIG file was generated using a Perl script, and was used for visualization of differentially methylated regions (DMRs). Similar workflows were followed to construct the DNA-seq library and PE150 sequencing. Clean reads were mapped to the reference genome using the Burrows-Wheeler-Alignment Tool (BWA) (Li and Durbin, 2009). The Genome Analysis Toolkit (GATK *v*.4.0) was used to analyze the SNPs and Insertion/Deletion mutations (InDels) throughout the birch genome (Poplin et al., 2018).

RNA was isolated from the fresh leaves (without petioles) of the same four individuals using the OminiPlant RNA Kit (DNase I), and RNA sequencing (RNA-seq) was performed on the Illumina HiSeq 2000 platform. Each sample was performed with three independent technical replicates. Raw transcriptome data was filtered using Fastp and the clean reads were mapped to the *B. pendula* reference genome using the HISAT2 aligner with default parameters (Kim et al., 2019). Expression of each gene was calculated in transcripts per million (TPM) by converting the sorted BAM alignment files with StringTie software (Pertea et al., 2015). Small RNA (sRNA) data was also aligned to the reference genome using BOWTIE software with parameters that allow up to one base mismatch. sRNA abundance for each region was calculated in reads per million (RPM) (Langmead et al., 2009). Summary statistics for the MethylC-seq, DNA-seq, RNA-seq and sRNA-seq libraries can be found in **Supplementary Tables S3**, **S4**.

### Quantitative reverse transcription PCR validation of gene expression

The relative expression levels of randomly-selected genes involved in lignin biosynthesis were verified *via* qRT-PCR using Sl, Fhs, Ame, and Alh samples. After RNA was extracted from leaves as described above, cDNA was synthesized through reverse transcription reactions. The total reaction mixture was 10 μL per sample and contained 1 μL Primer Script RT enzyme, 1 μL RT Primer Mix, 4 μL 5× Primer Script buffer, and 4 μL RNase-free ddH_2_O. The reaction conditions were set as follows: 15 min at 37°C, 5 s at 85°C, and 10 min at 0°C. The total reaction mixture for qRT-PCR was 15 μL, which contained 6.3 μL of 10× diluted cDNA template, 7.5 μL SYBR Green Supermix (catalog number: RK02001), 0.6 μL forward primer, and 0.6 μL reverse primer. Reactions were carried out in technical triplicate on the Applied Biosystems ABI7500 real-time PCR machine. The transcript abundance for each gene was calculated using the 2^-ΔΔCt^ method (Skern et al., 2005) with *tubulin* (*Bpev01.c0597.g0026*) used as the internal control for expression normalization. Primers used for qRT-PCR are shown in **Supplementary Table S5**.

### Statistical analysis

Lignin content for each birch was visualized in the study area of the northeast China using the R package ‘maptools’ (South, 2011). Spearman’s correlation was used to assess the relation between lignin content and tree age. To eliminate the potential interference of age factors, a partial correlation analysis was conducted to evaluate the relations between lignin content and climatic variables (Sadeghi and Marchetti, 2012). Correlations were considered significant at *p* ≤ 0.05. Differences in lignin content and gene expression between the two groups of birches were assessed with Student’s *t*-test, and DMRs that surrounded the relevant genes were analyzed with a hypergeometric test (Zhang et al., 2016). Significant differences were called at *p ≤* 0.05.

## Results

### Negative correlations between lignin content and temperature ranges

**Figure 1:**
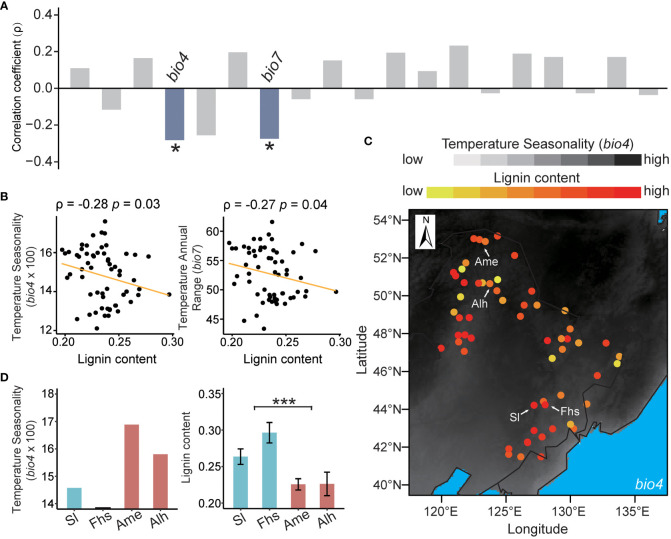
Correlations between lignin content and climatic variables. **(A)** Partial correlations between lignin content and 19 climatic variables. The correlation coefficients (ρ) in blue bar mean the lignin content is significantly negatively correlated with the indicated climatic variables, and those in grey mean the lignin content is not significantly correlated with the indicated climatic variables. **(B)** Distribution of lignin content with Temperature Seasonality (*bio4*) and Temperature Annual Range (*bio7*). The segmental line in orange represents the partial correlation fit of lignin to an indicated climatic variable. **(C)** Visualization of the relationship between lignin content and *bio4* in the northeast China. The color of the point in background reflects the value of *bio4*, with darker for a higher value and lighter for a lower value. The color of sampling sites reflects the content of lignin, with a redder color for a higher content and *vice versa*. The symbols “Sl”, “Fhs”, “Ame”, and “Alh” referred to the sample from different sampling sites, namely “Shulan”, “Fenghuangshan”, “Amuer”, and “Alihe”. **(D)** Comparisons of Temperature Seasonality (*bio4*) and the lignin content in Sl, Fhs, Ame, and Alh. The p ≤ 0.05 is marked as ‘*’, and p ≤ 0.001 is marked as ‘***’.

The lignin content of 60 natural birch individuals with a diverse geographic distribution ranged from 0.2 to 0.3 (**Supplementary Figure S1** and **Supplementary Table S1**). Spearman’s correlation analysis showed that the lignin content was independent of tree age (ρ = 0.22, *p* = 0.1) (**Supplementary Figure S2**). To control for the potential influence of tree age, partial correlations were used to analyze the relationships between the lignin content and 19 climatic variables. We found that the lignin content of natural birch was significantly negatively correlated with the local temperature ranges (**Figure 1A** and **Supplementary Table S6**). Specifically, the correlation coefficient between lignin content and Temperature Seasonality (*bio4*) was -0.28 (*p* = 0.03), and the correlation coefficient for Temperature Annual Range (*bio7*) was -0.27 (*p* = 0.04) (**Figure 1B**). These results suggested that temperature ranges may have a negative effect on the lignin content in native birch.

### Expression profiles of lignin biosynthesis genes and TFs

**Figure 2:**
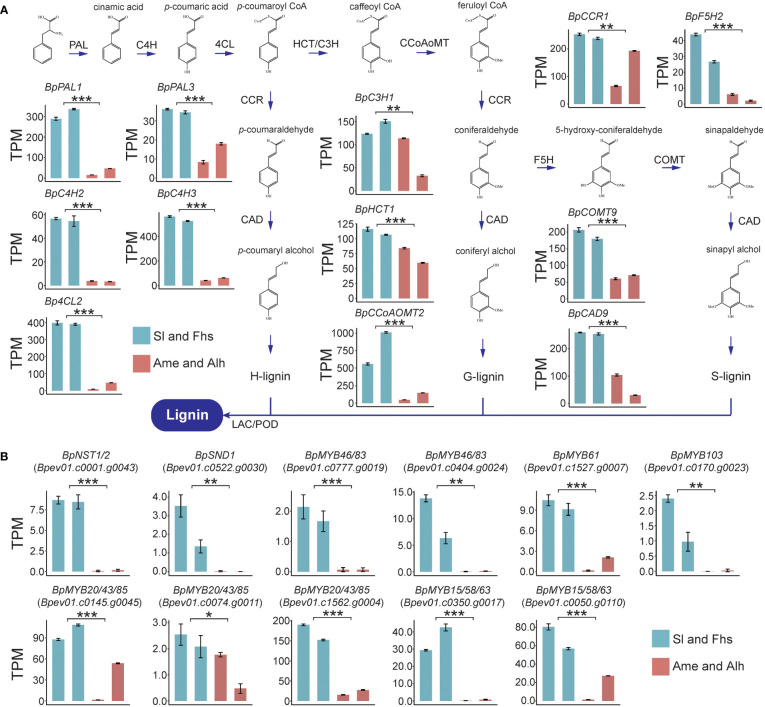
Expression profiles of lignin biosynthesis genes **(A)** and TFs **(B)** in Sl, Fhs, Ame, and Alh. Transcriptome levels for 12 lignin biosynthesis genes and 11 TFs were normalized to TPM (Transcripts Per Million). Colors represent the groups of birch trees (Sl and Fhs in green, and Ame and Alh in red). The *t*-test was used to evaluate the difference in gene expression between Sl, Fhs and Ame, Alh. The *p* ≤ 0.05 is marked as ‘*’, *p* ≤ 0.01 is marked as ‘**’, *p* ≤ 0.001 is marked as ‘***’.

To illustrate the molecular regulatory mechanisms affecting temperature ranges related lignin biosynthesis, the expression patterns of lignin biosynthesis genes of Sl, Fhs, Ame, and Alh were analyzed. Overall, Sl and Fhs (located in Changbai Mountain), which experience lower temperature ranges, had higher lignin content than Ame and Alh (located in Daxing Anling) (**Figures 1C, D** and **Supplementary Figures S3**, **S4**). The expression levels of all known genes (except for *BpCCR4*) involved in lignin biosynthesis were significantly reduced in birch (Ame and Alh) with lower lignin content compared to those (Sl and Fhs) with higher lignin content (**Figure 2A**, **Supplementary Figure S5** and **Supplementary Tables S7**–**9**). For example, *BpPAL1* (*Bpev01.c0042.g0040*), encoding the first enzyme of the general phenylpropanoid pathway catalyzing the deamination of phenylalanine (Higuchi et al., 1997), was expressed 10-fold lower in the Ame and Alh than in the Sl and Fhs (*p* = 3.58E-09). Similarly, *BpCOMT9* (*Bpev01.c0210.g0051*), that encodes a S-adenosylmethionine-dependent ortho-methyltransferase (Saluja et al., 2021), was expressed about three-fold lower in the Ame and Alh than in the Sl and Fhs individuals (*p* = 3.50E-07). Relative expression levels of several randomly-selected genes involved in lignin biosynthesis were further verified with qRT-PCR (**Supplementary Figure S6**). These results suggested that differences in the lignin content of natural birch may be related to the changes in the expression levels of lignin biosynthesis genes.

Numerous TFs, such as the MYB-domain and NAC-domain TFs, have been reported to directly or indirectly regulate the expression of multiple downstream lignin biosynthesis genes (Nakano et al., 2015; Ohtani and Demura, 2019; Xiao et al., 2021). Here, we used BLAST alignment and phylogenetic analysis of known *Arabidopsis* lignin biosynthesis regulators to identify 18 homologous TFs in birch, 14 of which were putative positive regulators and 4 of which were putative negative regulators. (**Supplementary Figure S7**, **Supplementary Table S7**). Among the 14 TFs that may positively regulate lignin biosynthesis in birch, we found that the expression levels of 11 were significantly reduced in birch individuals with lower lignin content (Ame and Alh) than in those with higher lignin content (Sl and Fhs) (**Figure 2B** and **Supplementary Tables S8** and **S9**). The remaining 3 putative positive regulators (*BpVND6/7*, *Bpev01.c0022.g000*5 and *Bepv01.c0411.g0006*; *BpMYB61*, *Bpev01.c0918.g0016*) were expressed at very low levels in all individuals. Notably, the expression level of *BpNST1/2* (*Bpev01.c0001.g0043*) and *BpSND1* (*Bpev01.c0522.g0030*), as the homologs of master controller of all three major secondary wall components in *Arabidopsis* (Fang et al., 2020), were significant decreased (*p* = 1.31E-07 and 2.68E-03, respectively) in birch with lower lignin content (Ame and Alh) than in those with the higher lignin content (Sl and Fhs), suggesting that the down-regulation of positively-regulated MYB-domain TFs and lignin biosynthesis genes may be subjected to these two NAC-domain TFs. In addition, the expression patterns of the 4 TFs that may negatively regulate lignin biosynthesis were not uniform (**Supplementary Figure S8**). For example, *BpMYB3*/*4*/*7*/*32* (*Bpev01.c0639.g0015*) was expressed at lower levels in Ame (*p* = 5.37E-03), but *BpMYB3*/*4*/*7*/*32* (*Bpev01.c0668.g0007*, and *Bpev01.c0549.g0004*) showed an enhanced expression (*p* = 5.60e-06 and 1.75E-04, respectively). Taken together, these results suggested that changes in the expression of lignin biosynthetic genes and of positively-regulated MYB-domain TFs (and thus in lignin content) are likely subject to expression of the two NAC-domain TFs, *BpNST1/2* and *BpSND1*.

### Correlations of *BpNST1/2* and *BpSND1* expression with DNA methylation

**Figure 3:**
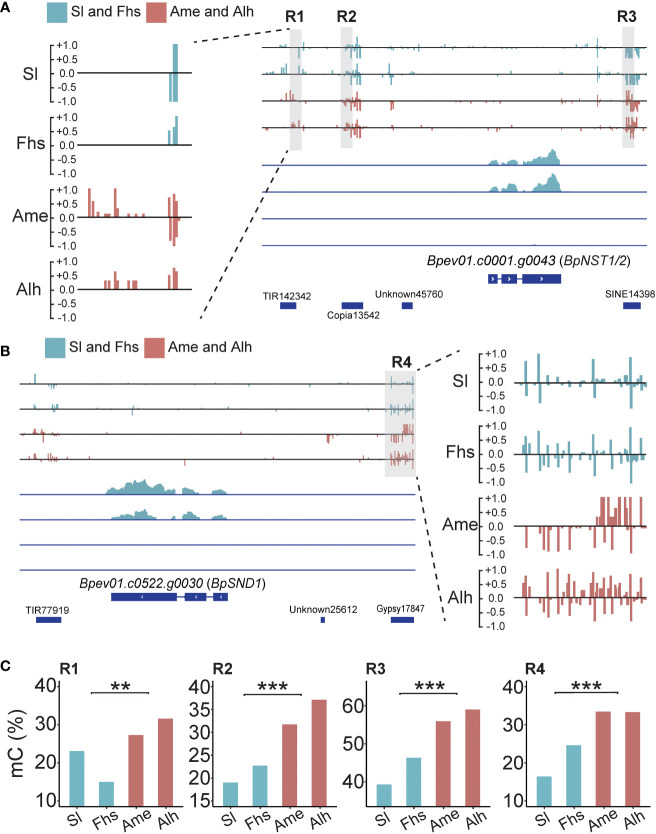
Methylation and expression profiles of *BpNST1/2* and *BpSND1*. **(A)** Methylation and transcript snapshot of *BpNST1/2* in Sl, Fhs, Ame, and Alh. R1 (Chr5:20,594,500-20,594,600), R2 (Chr5:20,595,495-20,595,599) and R3 (Chr5:20,600,700-20,600,825) marked in grey box were identified as the DMRs nearby the *BpNST1/2.* The height of each column represents the methylation level at each cytosine. The lower part indicates the transcript level of *BpNST1/2*, ranging from 0 to 100. **(B)** Methylation and transcript snapshot of *BpSND1* in Sl, Fhs, Ame, and Alh. R4 (Chr14:277,350-277,600) marked in grey box was identified as the DMRs located in promoter of *BpSND1.* The lower part indicates the transcript level of *BpSND1*, ranging from 0 to 30. **(C)** DNA methylation level of R1-R3 (*BpNST1/2*) and R4 (*BpSND1*) in Sl, Fhs, Ame, and Alh. The p ≤ 0.01 is marked as ‘**’ and p ≤ 0.001 is marked as ‘***’".

Previous studies have shown that DNA methylation variations caused by temperature changes may affect nearby gene expressions (Naydenov et al., 2015; He et al., 2018; Zhu et al., 2020). Here, we found four DMRs: three near *BpNST1/2* (R1, R2 and R3) and one near *BpSND1* (R4). All four had higher methylation levels in Ame and Alh than those in Sl and Fhs (**Figure 3**, **Supplementary Figure S9** and **Supplementary Table S10**). For instance, the R1 region (Chr5:20,594,500-20,594,600) located on a TE (TIR142342) 3.5 kb upstream of *BpNST1/2*, where the weighted methylation level in Sl and Fhs (18.18%) was significantly lower than that in Ame and Alh (29.10%, *p* = 1.13E-03). Similarly, the R4 region (Chr14:277,346-277,605) located on a TE (Gypsy17847) 2.0 kb upstream of *BpSND1*, where the weighted methylation level in Sl and Fhs (20.51%) was significantly lower than that in Ame and Alh (33.36%, *p* = 1.47E-146). These results showed an opposite trend compared to the expression patterns observed for *BpNST1/2* and *BpSND1*, suggesting that hypermethylation in the promoter regions of *BpNST1/2* and *BpSND1* may contribute to the repression of these genes expression.

### CHH methylation of *BpNST1/2* and *BpSND1*

**Figure 4:**
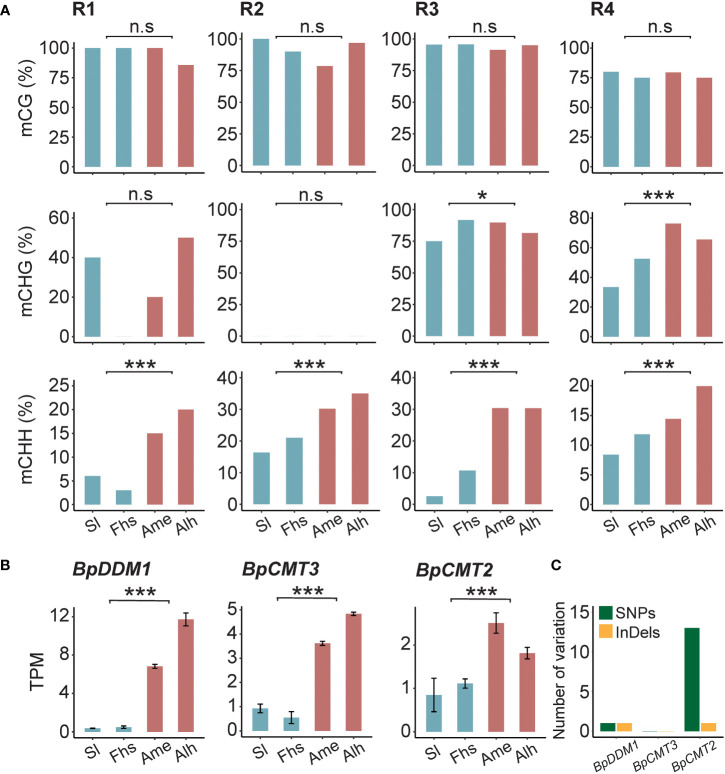
DNA methylation of *BpNST1/2* and *BpSND1* in different sequence background. **(A)** CG, CHG and CHH methylation levels in the R1-R3 (*BpNST1/2*) and R4 (*BpSND1*), and **(B)** transcript level of *BpDDM1*, *BpCMT3* and *BpCMT2* in Sl, Fhs, Ame, and Alh. The p ≤ 0.05 is marked as ‘*’, p ≤ 0.001 is marked as ‘***’ and p > 0.05 is marked as ‘n.s’. **(C)** Number of nonsynonymous mutations in the introns of *BpCMT2*, *BpCMT3* and *BpDDM1*. Genetic variants were counted when variants at the locus were completely distinct from Ame and Alh to Sl and Fhs.

To assess whether the methylation patterns of *BpNST1/2* and *BpSND1* differed in sequence background, CG, CHG, and CHH methylation levels in the R1-R4 regions were calculated, respectively. We found that the CHH methylation levels of these 4 regions varied significantly between the two groups of birch, consistent with the trend of total methylation (**Figures 4A**, **3C** and **Supplementary Tables S10**, **S11**). However, there was no such observations in CG methylation. For example, for the R1 region of *BpNST1/2*, the weighted CG methylation level of birch in Sl and Fhs (1.0) was not significantly different from that of Ame and Alh (0.94) (*p* = 5.29E-01). In contrast, Ame and Alh showed a significant increase in weighted CHH methylation (0.04 vs. 0.18, *p* = 2.84E-04). Small interfering RNA (siRNA) analysis indicated that CHH methylation variations of these 4 regions among different individuals were not correlated with the abundance of 24-nt siRNA (**Supplementary Figure S10** and **Supplementary Table S12**), suggesting that the RdDM pathway may *de novo* establish the DNA methylation of these regions, but the maintenance of the DNA methylation subject to mitosis or meiosis is somehow affected by the natural environments (Elhamamsy, 2016; Johannes and Schmitz, 2019). Furthermore, the transcriptomes showed that the expression levels of three critical genes (*BpCMT2*, *BpCMT3* and *BpDDM1*) involved in CHH methylation maintenance were significantly up-regulated in Ame and Alh, compared with those in Sl and Fhs (**Figure 4B**). Although we did not find homozygous non-synonymous mutations in the exons of these three genes between the two groups birch (Sl and Fhs vs. Ame and Alh), there are 14 (13 snp and 1 indel) and 2 (1 snp and 1 indel) potential natural selective genetic variants in the introns of *BpCMT2* and *BpDDM1*, respectively (**Figure 4C** and **Supplementary Table S13**). In conclusion, we hypothesized that temperature ranges, such as Temperature Seasonality, may influence lignin biosynthesis by affecting the maintenance of non-CG methylation in the promoter regions of the *BpNST1/2* and *BpSND1*.

## Discussion

Lignin is a group of phenolic polymers which is abundant in the woody tissues of vascular plants and has long been considered to play an important role in plant growth and development (Liljegren et al., 2000; Bonawitz et al., 2014; Peng et al., 2014). Recently, a few studies have shown that the lignin biosynthesis is related to plant adaptations to external stresses, such as metal exposure, drought, salinity, and temperature stresses (Mao et al., 2004; Moura-Sobczak et al., 2011; Liu et al., 2018). For example, Gindl et al. (2000) found that lignin deposition in the late terminal trachea of *Norway spruce* is positively correlated with temperature from early September to late October. Yun et al. (2013) found that high temperature stress can induce lignin deposition in *Satsuma mandarin* pericarp. Similarly, at 30°C, the accumulation of lignin in the MtCAD1 mutant of *Medicago* was inhibited and thus the vascular system was damaged, which led to impaired transpiration and overheating and thus the stalled growth (Zhao et al., 2013). In addition, an increased lignin production during cold acclimation has also been observed in several species, suggesting a physiological mechanism of freezing tolerance in trees (Griffith et al., 1985; Marzanna et al., 1999; Wei et al., 2006; Vitasse et al., 2014). Besides environmental factors, tree age is also shown to affect lignin content (Durkovic et al., 2012). However, the general trend is not consistent and somehow controversy with increase (Jahan and Mun, 2005; Rencoret et al., 2011), decrease (Stolarski et al., 2011; Healey et al., 2016) or no responses (Lachowicz et al., 2019) of lignin in wood being reported with tree aging. In this study, we found that the impact of the age of trees on lignin content was not significant (**Supplementary Figure S2**). This inconsistency among various studies may be ascribed to other factors, including the species, whether the wood came from a plantation or a natural forest, forest habitat type, the part of the tree from which samples were taken, the time of sampling, etc. (Lachowicz et al., 2019). Nevertheless, after controlling for the potential influence of tree age, we found that the lignin content of natural birch was significantly negatively correlated with the temperature ranges (*bio4* and *bio7*) (**Figure 1** and **Supplementary Table S6**). This suggested that temperature regimes can play a significant role in lignin biosynthesis, and that changes in lignin content may contribute to plants adaptations to fluctuations in temperature.

Uncovering the basis underlying the associations between traits and the environment is a central topic in molecular biology. At the molecular level, DNA methylation is thought to be involved in plant plasticity in the face of environmental stresses and/or climate changes, but solid evidence in natural population is few and mostly come from *Arabidopsis*. For example, the global DNA methylation of *Arabidopsis* accessions are negatively associated with the temperatures in Europe, in which the accessions with hypomethylomes are enriched in habitats with higher temperatures (Kawakatsu et al., 2016). The methylation level of NMR19, a naturally occurring epiallele controlling leaf senescence, is negatively associated with the Mean Temperature of Driest Quarter (*bio9*) (He et al., 2018). As one of the studies that focuses on trees, Wang et al. (2021) found that in natural birch the methylation variations of *bHLH9* are associated with winter temperature and summer precipitation and affected betulin biosynthesis. Overall, these results suggested that temperature regimes are strong environmental factors that can shape the methylation patterns of key genes along their gradients. To support this, we found that DNA methylation levels of the two NAC-domain TFs (*BpNST1/2* and *BpSND1*) were significantly correlated with the local Temperature Seasonality (*bio4*) and Temperature Annual Range (*bio7*) (**Figures 1** and **3**). These trends were strong, especially in CHH sequence context (**Figure 4A**). This result was consistent with the temperature-dependent characteristics of CHH methylation (Bewick and Schmitz, 2015), which probably caused by the differentiation of *CMT2* alleles that participates the maintenance of CHH methylation status along temperature regime gradients, such as temperature (Dubin et al., 2015) and Temperature Seasonality (Shen et al., 2014). Indeed, we found 14 genetic differences in the intron of *BpCMT2* between low and high temperature ranges regions (**Figure 4C** and **Supplementary Table S13**). These differences may form alleles and lead to different expression patterns of *BpCMT2* or alter the splicing of mRNA. Additionally, there were significant expression differences of *BpCMT3* and *BpDDM1* but no and only 2 genetic variations in intron, respectively, between low and high temperature ranges regions (**Figures 4B, C** and **Supplementary Table S13**). Nevertheless, other factors, such as environmentally-induced DNA methylation (Miryeganeh et al., 2022), may also contribute to the differential expressions of *BpCMT2*, *BpDDM1* and *BpCMT3*.

One of the crucial consequences of DNA methylation is to affect the gene expressions and thus contribute to phenotypic variations (Zemach et al., 2013; Zhang et al., 2018). Several epigenetic variations with phenotypic consequences have been characterized as epialleles, such as plant architecture (Miura et al., 2009), flowering time (Ikeda et al., 2007), root length (Cortijo et al., 2014), and biomass (Ong-Abdullah et al., 2015). Although limited epialleles have been identified so far, transposons in which most epigenetic variations occur may be important carriers for epialleles (Slotkin and Martienssen, 2007). In birch, the content of transposons can be up to 50% with a relatively uniformly distribution in the genome, suggesting that this species is prone to have more epialleles (Salojarvi et al., 2017; Wang et al., 2021). To support this, we found that both differential methylation modifications of *BpNST1/2* and *BpSND1* occurred on transposons (TIR and Gypsy, respectively) within the gene promoter region and affected corresponding gene expressions (**Figures 3A, B**). Similarly, other genes, such as *Bp4CL2*, *BpCOMT9*, and *BpMYB103*, also differed significantly in their promoter (2 kb upstream of ATG) DNA methylation, which occurred on TEs (Copia, TIR and TIR) (**Supplementary Figure S11** and **Supplementary Table S14**). These methylation variations were also opposite to their genes’ expression pattern (**Figure 2**). Thus, we cannot exclude the possibility that the DNA methylation modification of these genes’ promoter could be involved in regulating their expression. Yet, we believe that the differentiation of DNA methylation and the resultant gene expression patterns of *BpNST1/2* and *BpSND1*, which are the first-layer switches in regulation of downstream lignin biosynthesis genes and MYB-domain TFs (Zhong et al., 2007; Golfier et al., 2017; Fang et al., 2020), are likely to be the main cause of the changes in these downstream genes’ expression and thus lignin biosynthesis. Further, we found that CHH and part of CHG methylation levels on transposons of *BpNST1/2* and *BpSND1* were negatively related to their expressions (**Figures 2B**, **4A**), similar to the CMT2/3-dependent epiallele (*Cnr*) that regulates tomato fruit ripening in tomato (Chen et al., 2015). Likely, we recently found that CHH methylation can be involved in anthocyanin biosynthesis (another downstream branch of the phenylpropane metabolic pathway) by affecting the expression of *CmMYB6* in chrysanthemum (Tang et al., 2022). We hypothesized that CHH as well as CHG methylation can form a heterochromatin by coupling with the H3K9me2, thereby inhibiting the gene expression by restricting the access of TFs to their binding sites in promoters, which may ultimately result in the changes of lignin content. This positive feedback loop between H3K9me2 and non-CG methylation has also been considered as a major contributing factor to the origins of spontaneous epialleles and that heterochromatin is a quantitative trait that influences epiallele formation (Zhang et al., 2021).

Cellulose and hemicellulose are also the target products regulated by *BpNST1/2* and *BpSND1*. We evaluated the transcript levels of critical cellulose and hemicellulose biosynthesis genes (10 homologs of *BpCesA* and 11 homologs of *BpCsl*) in Sl, Fhs, Ame, and Alh (**Supplementary Figure S12** and **Supplementary Table S15**). The gene expression levels of *BpCesA04*, *BpCesA07*, and *BpCesA08*, which function mainly in the secondary plant cell wall (Desprez et al., 2007; Persson et al., 2007), were significantly decreased in Ame and Alh (trees with high temperature ranges) compared with Sl and Fhs (trees with low temperature ranges), and this expression pattern was highly consistent with the expression of *BpSND1* (**Figure 2B**). Further, the expression level of *BpCslB* and *BpCslG* subfamilies, which are specific in non-grass angiosperms (Keegstra and Walton, 2006; Suzuki et al., 2006), were tightly correlated with that of *BpNST1/2* and *BpSND1* (**Supplementary Figure S13** and **Supplementary Table S15**). Based on these findings, we hypothesised that these two gene families (*CesA* and *Csl*) may be commonly indirectly regulated by *NST1/2* and *SND1* in trees, and likely play a role in the biosynthesis of cellulose and hemicellulose in second cell wall of woody plants.

**Figure 5:**
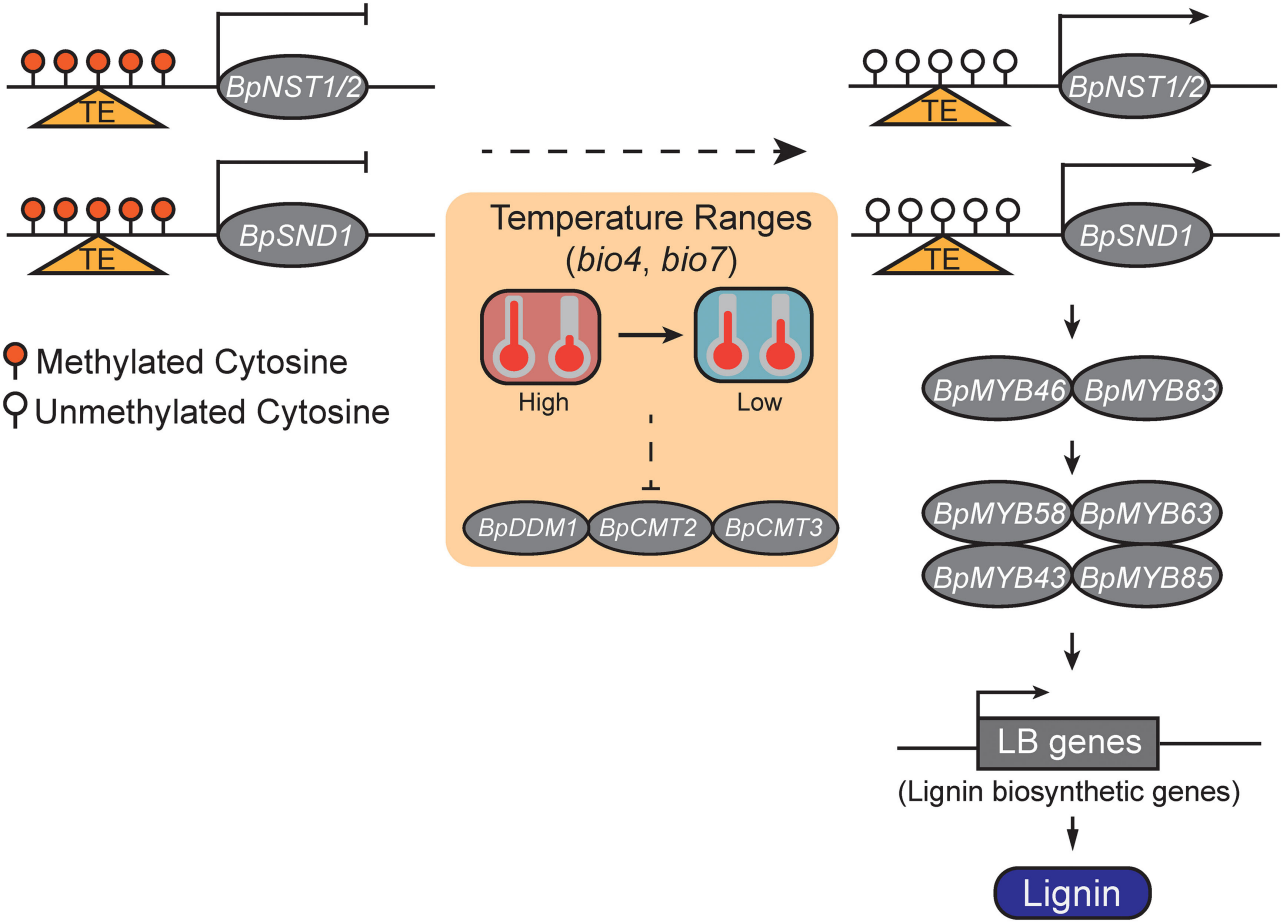
A schematic model of temperature ranges (TRs) responsive DNA methylation involved in lignin biosynthesis *via* the regulation of the expression of *BpNST1/2* and *BpSND1*. The small red balloons represent highly methylated cytosines, white ones represent unmethylated cytosines, and the triangles in orange indicate a genomic region with transposable element (TE) insertion. With a decreasing of TRs, the methylation levels of the promoter region of the *BpNST1/2* and *BpSND1* decrease and their expressions are thus enhanced, leading to the up-regulation of downstream MYB-domain TFs and lignin biosynthesis genes and, ultimately, increasing the content of lignin in birch.

To summary, we proposed a DNA methylation-mediated lignin biosynthesis model which responds to environmental factors (**Figure 5**). Specifically, relatively low temperature ranges may decrease DNA methylation levels of *BpNST1/2* and *BpSND1*, and lead to the enhanced expressions of *BpNST1/2* and *BpSND1* and the downstream MYB-domain TFs and lignin biosynthesis genes, and ultimately, result in a high level of lignin content. However, manipulated experiments are needed to test whether DNA methylation of *BpNST1/2* and *BpSND1* can be directly altered by Temperature Seasonality and/or Temperature Annual Range (TAR), and if variation of these methylation is heritable.

## Data availability statement

The datasets generated for this study are available on request to the corresponding authors. The sequencing data are available through the NCBI Sequence Read Archive under the accession number PRJNA902946.

## Author contributions

BC, JW, SS, and QZ conducted the experimental design, data analysis, and prepared the manuscript. YG, XZ, LW, TZ, LC, ZZ and WZ provided help in carrying out the experiments and guidance in designing the research. QZ, CY, SS, JW, LX and BC interpreted the results and modified the manuscript. All authors contributed to the article and approved the submitted version.

## Funding

This work was supported by the National Key R&D Program of China during the 14th Five-year Plan Period (2021YFD2200304), Heilongjiang Touyan Innovation Team Program (Tree Genetics and Breeding Innovation Team), the National Natural Science Foundation of China (31871220, 62273086, 62001088), the National Nonprofit Institute Research Grant of the Chinese Academy of Forestry (CAFYBB2019ZY003), and the Fundamental Research Funds for the Central Universities (2572022BD04, 2572018AA27).

## Conflict of interest

The authors declare that the research was conducted in the absence of any commercial or financial relationships that could be construed as a potential conflict of interest.

## Publisher’s note

All claims expressed in this article are solely those of the authors and do not necessarily represent those of their affiliated organizations, or those of the publisher, the editors and the reviewers. Any product that may be evaluated in this article, or claim that may be made by its manufacturer, is not guaranteed or endorsed by the publisher.

## Supplementary material

Supplementary Figure 1 Sampling sites of naturally growing birch in the northeast China. Each dot in orange indicates the sampling site. Accessions 1 to 19 (except 4 and 5) belong to Changbai Mountain; 24 to 35 belong to Xiaoxing Anling; 36 to 59 (including 4 and 5) belong to Daxing Anling; 20 to 23 belong to Sanjiang Plain.

Supplementary Figure 2 Correlation analysis between tree age and lignin content. Spearman’s correlation analysis was used to assess the relationship between the tree age and lignin content.

Supplementary Figure 3 Comparisons of Temperature Annual Range (*bio7*) in Sl, Fhs, Ame and Alh.

Supplementary Figure 4 Visualization of the relationship between lignin content and Temperature Annual Range (*bio7*) in the northeast China. The color of the point in the background reflects the value of *bio7*, with darker for a higher value and lighter for a lower value. The color of sampling sites reflects the lignin content, with a redder color for a higher content and *vice versa*.

Supplementary Figure 5 Transcript level of the *BpCCR4*. The p ≤ 0.001 is marked as ‘***’.

Supplementary Figure 6 Relative expressions of randomly-selected genes assessed with qRT- PCR. The *t*-test was used to evaluate the difference in genes expression between the two groups of birch trees. *p* ≤ 0.01 is marked as ‘**’ and *p* ≤ 0.001 is marked as ‘***’.

Supplementary Figure 7 Phylogenetic analysis of NAC-domain and MYB-domain TFs involved in lignin biosynthesis of birch and *Arabidopsis*. The rooted neighbor-joining (NJ) phylogenetic tree of TFs was clustered with bootstrap values shown for each clade in circles with a different size. Genes in bold with black circle outside is for the candidate genes that most probably encode phenylpropanoid biosynthesis enzymes.

Supplementary Figure 8 Transcript level of *BpMYB3/4/7/32*. The p ≤ 0.05 is marked as ‘*’, p ≤ 0.01 is marked as ‘**’ and p > 0.05 is marked as ‘n.s’.

Supplementary Figure 9 Snapshots of DNA methylation of R2 and R3 in Sl, Fhs, Ame, and Alh. The height of each column represents the methylation level at each cytosine.

Supplementary Figure 10 Snapshots of **(A)** CHH methylation and **(B)** 24nt-siRNA of R1-R4 in Sl, Fhs, Ame, and Alh. Of the methylation snapshot, the height of each column represents the methylation level at each cytosine in CHH context. Of the 24nt-siRNA snapshot, the height of each column represents the RPM of 24nt-siRNA in 10 bp window. **(C)** The bar chart indicates the RPM of 24nt-siRNA for each region (R1-R4).

Supplementary Figure 11 Promoter DNA methylation level of lignin biosynthesis genes and TFs in birch. **(A)** Heatmap of DNA methylation levels in the promoter regions (2kb upstream of ATG) of genes involved in lignin biosynthesis. **(B)** Methylation snapshot of *Bp4CL2*, *BpCOMT9* and *BpMYB103* in Sl, Fhs, Ame, and Alh. Box in grey color indicate regions with pronounced methylation variations in their promoter region.

Supplementary Figure 12 Transcript level of *BpCesA*s in birch. **(A)** Phylogenetic analysis of CesAs of birch and *Arabidopsis*. The rooted neighbor-joining (NJ) phylogenetic tree of CesAs was clustered with bootstrap values shown for each clade in circles with a different size. The *CesA*s of *Arabidopsis* with green star outside function mainly in second plant cell wall, and those with blue star outside function mainly in primary plant cell wall. “?” indicates the function of the gene is remain unclear. **(B)** Transcript level of *BpCesA*s in Sl, Fhs, Ame, and Alh. The *t*-test was used to evaluate the difference in genes expression between the Sl, Fhs and Ame, Alh. The *p* ≤ 0.01 is marked as ‘**’, *p* ≤ 0.001 is marked as ‘***’, and *p* > 0.05 is marked as ‘n.s’.

Supplementary Figure 13 Transcript level of *BpCsl*s in birch. **(A)** Phylogenetic analysis of Csls of birch and *Arabidopsis*. The rooted neighbor-joining (NJ) phylogenetic tree of Csls was clustered with bootstrap values shown for each clade in circles with a different size. Birch genes with black circle outside is for the candidate genes that most probably encode Csls in birch. **(B)** Transcript pattern of *BpCsl*s in Sl, Fhs, Ame, and Alh.
