# Supplementary figures and images for: Climate-responsive DNA methylation is involved in the biosynthesis of lignin in birch
Source: Front Plant Sci. 2022 Dec 2;13:1090967. doi: 10.3389/fpls.2022.1090967 (PMC9757698; doi:10.3389/fpls.2022.1090967)

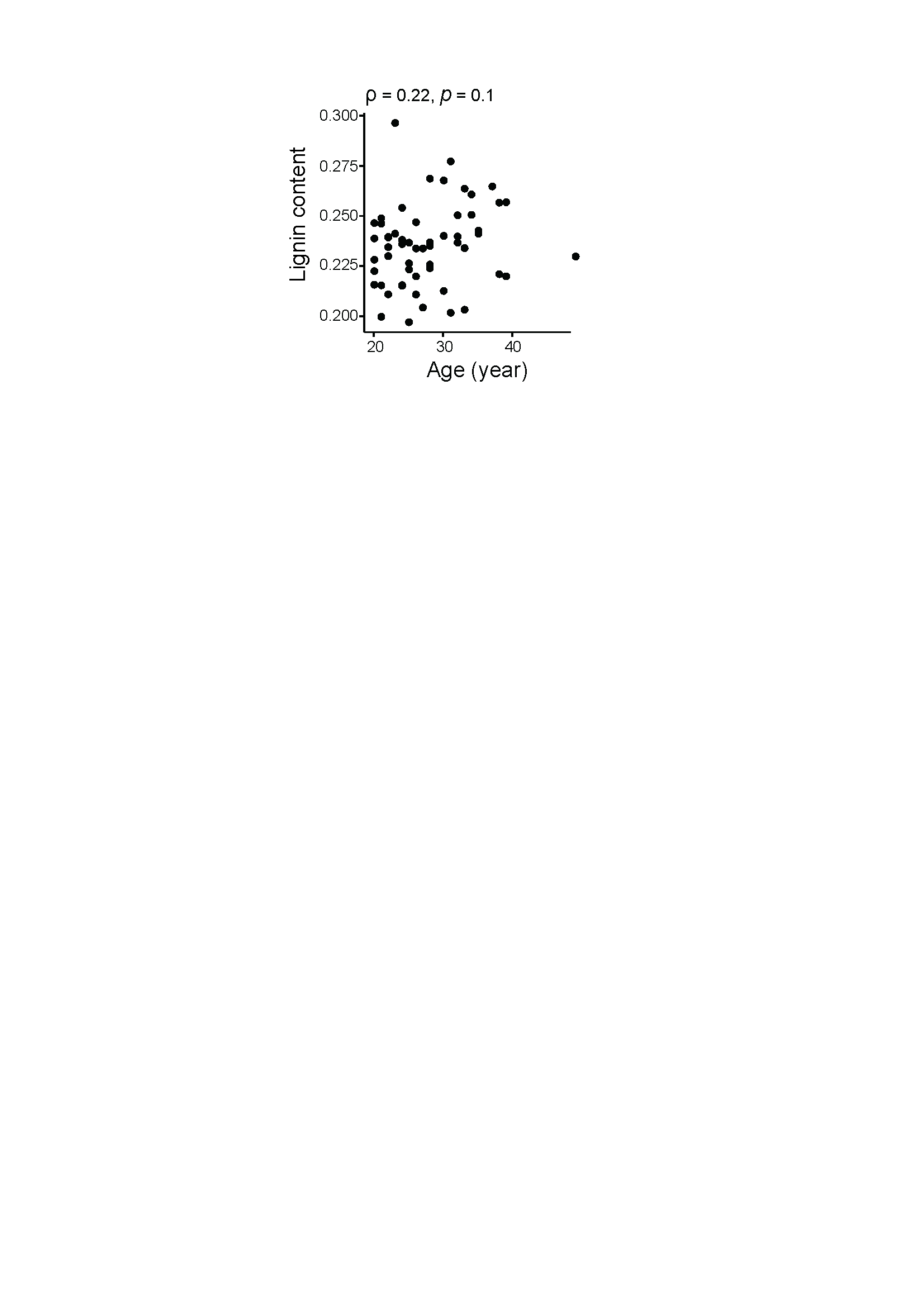

Supplement: Supplementary Figure 2 — Correlation analysis between tree age and lignin content. Spearman’s correlation analysis was used to assess the relationship between the tree age and lignin content. [file Image_2.tif]

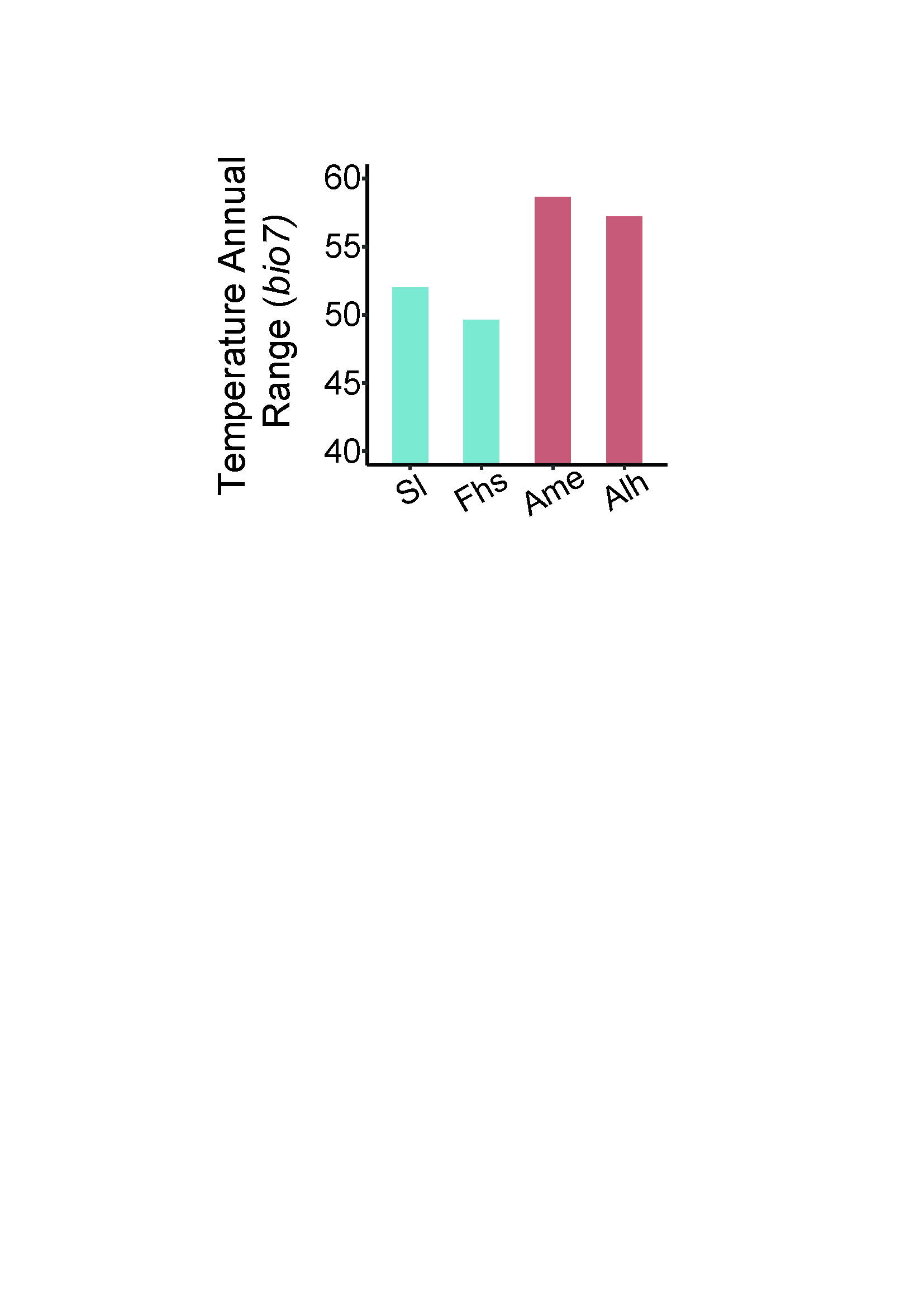

Supplement: Supplementary Figure 3 — Comparisons of Temperature Annual Range (bio7) in Sl, Fhs, Ame and Alh. [file Image_3.tif]

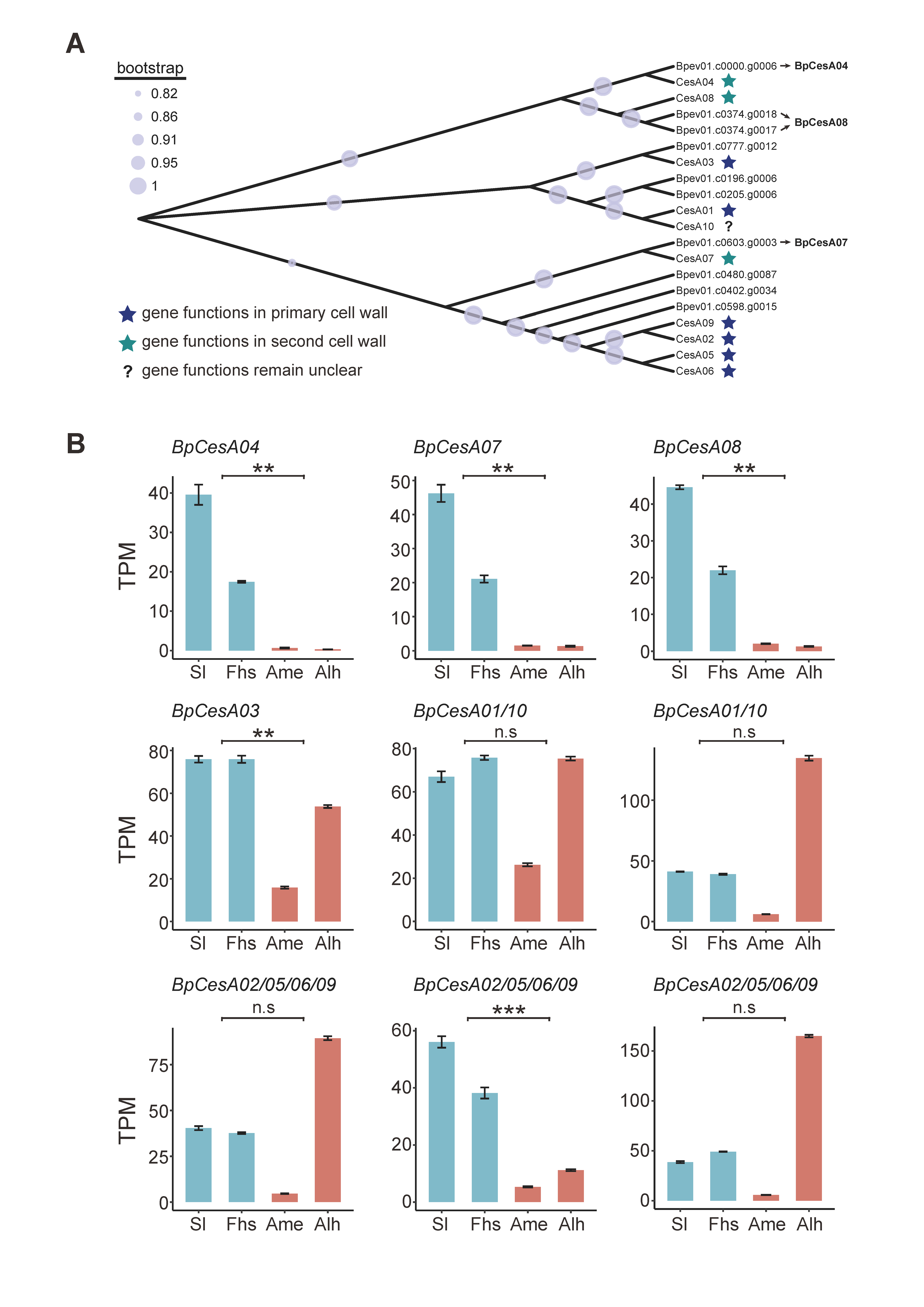

Supplement: Supplementary Figure 12 — Transcript level of BpCesAs in birch. (A) Phylogenetic analysis of CesAs of birch and Arabidopsis. The rooted neighbor-joining (NJ) phylogenetic tree of CesAs was clustered with bootstrap values shown for each clade in circles with a different size. The CesAs of Arabidopsis with green star outside function mainly in second plant cell wall, and those with blue star outside function mainly in primary plant cell wall. “?” indicates the function of the gene is remain unclear. (B) Transcript level of BpCesAs in Sl, Fhs, Ame, and Alh. The t-test was used to evaluate the difference in genes expression between the Sl, Fhs and Ame, Alh. The p ≤ 0.01 is marked as ‘**’, p ≤ 0.001 is marked as ‘***’, and p > 0.05 is marked as ‘n.s’. [file Image_12.tif]
